# Physical imaging phantoms for simulation of tumor heterogeneity in PET, CT, and MRI: An overview of existing designs

**DOI:** 10.1002/mp.14045

**Published:** 2020-02-12

**Authors:** Alejandra Valladares, Thomas Beyer, Ivo Rausch

**Affiliations:** ^1^ QIMP Team Centre for Medical Physics and Biomedical Engineering Medical University of Vienna Vienna 1090 Austria

**Keywords:** CT, MRI, PET, phantom, tumor heterogeneity

## Abstract

**Background:**

In oncology, lesion characterization is essential for tumor grading, treatment planning, and follow‐up of cancer patients. Hybrid imaging systems, such as Single Photon Emission Computed Tomography (SPECT)/CT, Positron Emission Tomography (PET)/CT, or PET/magnetic resonance imaging (MRI), play an essential role for the noninvasive quantification of tumor characteristics. However, most of the existing approaches are challenged by intra‐ and intertumor heterogeneity. Novel quantitative imaging parameters that can be derived from textural feature analysis (as part of radiomics) are promising complements for improved characterization of tumor heterogeneity, thus, supporting clinically relevant implementations of personalized medicine concepts. Nevertheless, establishing new quantitative parameters for tumor characterization requires the use of standardized imaging objects to test the reliability of results prior to their implementation in patient studies.

**Methods:**

In this review, we summarize existing reports on heterogeneous phantoms with a focus on simulating tumor heterogeneity. We discuss the techniques, materials, advantages, and limitations of the existing phantoms for PET, CT, and MR imaging modalities.

**Conclusions:**

Finally, we outline the future directions and requirements for the design of cross modality imaging phantoms.

## 1 Introduction

In medical imaging, physical phantoms refer to real objects designed to simulate the human body, or parts of it, for specific clinical conditions. Physical phantoms are used to calibrate imaging systems, to evaluate their performance and to ensure the correct operation of imaging systems before scanning human subjects.^1^ They also constitute an inexpensive way of testing new imaging applications and serve as a well‐defined reference for quantitative measurements.

In oncology, four principal tomographic imaging modalities are used in clinical routine for the diagnosis and characterization of malignancies: single photon emission computed tomography (SPECT), positron emission tomography (PET), CT, and magnetic resonance imaging (MRI). For each of these modalities, numerous phantoms have been developed and employed across imaging sites to evaluate performance. These phantoms are available in a wide range of shapes and functionalities, from simple water‐filled cylinders to more sophisticated three‐dimensional (3D) printed anthropomorphic objects.^2^ Moreover, specific phantoms exist for hybrid imaging systems such as PET/CT and PET/MRI, catering to the special needs of each imaging component.^3^

Hybrid imaging systems play an important role in oncology. The combined imaging modalities provide complementary morphological and physiological information within a single examination, thus, improving diagnostic accuracy and subsequently patient management.^4^, ^5^, ^6^, ^7^ Since tumor characterization is critical for the diagnosis/grading, treatment planning, and follow‐up of the oncological patients, different methods aim at quantitating the characteristics of the tumor. Tumor length (e.g., by applying the Response Evaluation Criteria in Solid Tumors, RECIST^8^), tumor volume, standardized uptake values (SUV) calculated from PET images, and tissues elasticity measured by MRI, are some examples of commonly used quantitation parameters.^9^, ^10^ However, most of these parameters are challenged by intra‐ and intertumor heterogeneity. Therefore, textural feature extraction and assessment through radiomics have been proposed as a promising means for a more encompassing characterization of lesions and lesion heterogeneity.^11^, ^12^

Nevertheless, the reproducibility and comparability of radiomics features is still a matter of debate,^13^, ^14^, ^15^, ^16^, ^17^ and standardization efforts are yet to be expanded. Therefore, optimum standardized imaging objects along with imaging and analysis procedures are needed for facilitating the definition of benchmark parameters for pathology‐specific imaging applications. Previous studies for different imaging systems have reported on phantoms that recapitulate the heterogeneous anatomical regions of human physiology.^18^, ^19^, ^20^, ^21^, ^22^, ^23^ Nonetheless, most of the existing phantoms for SPECT, PET, CT, and MRI are not suitable for multimodality imaging. They are limited in their abilities to repeat and reproduce the heterogeneous nature of the tumor. In summary, the design and development of such phantoms are challenging, particularly for multimodality imaging systems and multicenter studies.

This review highlights ongoing efforts on designing suitable heterogeneity phantoms for advanced image quality assessment, which ultimately can be used for broad adoption of describing intra‐/interlesion heterogeneity in different imaging setups. We focus on the techniques, materials, advantages, and limitations of the existing phantoms for MR, CT, PET, and PET/CT imaging systems. Furthermore, current directions and requirements for the design of cross modality imaging phantoms will be outlined. At this stage, we shall not include SPECT phantoms, given the insignificant number of reports dedicated to evaluating tumor heterogeneity with SPECT. Furthermore, physical requirements for SPECT imaging phantoms are very similar to those for PET, and therefore the section on PET phantoms can be used as a guidance to design relevant SPECT phantoms.

## 2 MR Imaging phantoms

MR imaging is based on the measurement of electromagnetic radiation originating from the realignment of excited nuclear magnetic moments. In clinical MR imaging, the magnetic moments of hydrogen atoms are targeted due to their abundance in biological tissue, therefore, resulting in appropriate signal intensities. The excellent soft tissue contrast achieved with MRI is due to the differences in relaxation times of different tissues. However, if the relaxation times are too short, as it is the case in most solid materials, MRI systems will not produce sufficiently high signals for detection. Therefore, most of the existing MRI phantoms are composed of tissue‐mimicking materials, such as water, fat, and agarose gel.^24^, ^25^, ^26^ In terms of phantom building techniques and materials, the design criteria for quantitative MRI phantoms are well‐characterized MRI properties and stability of the used materials. It is also recommended avoiding fillable compartments to ensure traceability and consistency of the phantom characteristics. Furthermore, it is suggested to have phantom components which have stability for at least 5 yr, especially for multicenter use.^27^ In the matter of the simulation of tumor heterogeneities, there are only a few reports found in the literature, which are presented below.

**Figure 1:**
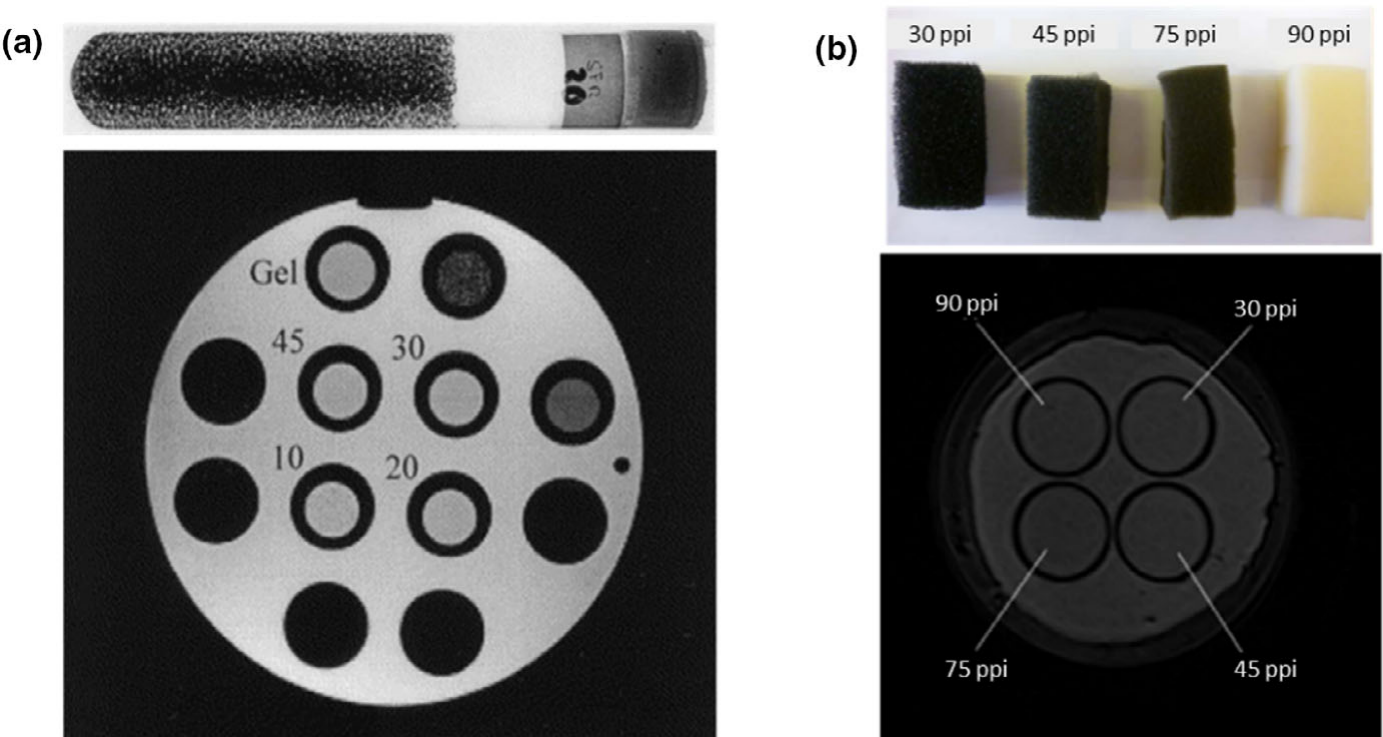
(a) Texture phantom proposed by Lerski and Schad (1998). Top: test tube with reticulated foam embedded in agarose gel. Bottom: axial view of the phantom. The numbers represent the pores sizes used, 10 being the bigger and 45 the smaller one. (b) Top: Reticular foam inserts embedded in a cylindrical phantom filled with agarose gel as proposed by Waugh et al. Bottom: phantom axial view (ppi: pores per inch). Adapted from Fig. 1 in Lerski et al.^28^ and Fig. 2 in Waugh et al.^29^ [Color figure can be viewed at http://wileyonlinelibrary.com]

Lerski and Schad reported first on building heterogeneity phantoms for MR imaging.^28^ The authors used porous foams embedded in agarose gel and then inserted in Pyrex test tubes for the modeling of structural differences. For comparison, they also use a tube filled only with gel, and a tube filled with glass beads (1 mm diameter). All the tubes were placed in a cylindrical phantom [Fig. 1(a)]. Phantom images were acquired in a Siemens 1 T whole‐body MRI system with a conventional head coil. Texture analysis and principal component analysis were performed on the acquired images. Results showed that the best clustering was reached for the foam with the bigger pores size. For the other foams, the separation was less clear. The authors recommended the use of these objects for calibration of in vivo texture measurements.

The use of porous foams for texture analysis of MR phantom images was also reported in another study.^29^ Here, a breast‐mimicking phantom was built to evaluate the reliability of textural features when using different clinical breast MRI protocols. Different test tubes were filled with a 2% agarose solution doped with 0.2% gadolinium to reproduce T1 relaxation times that are typically observed in clinical studies. Foams with different pore sizes were submerged in the tubes, and were then placed in a cylindrical container filled with the same 2% agarose solution. Phantom image acquisitions were performed in a 1.5 and 3 T MRI systems with a standard breast coil. An example of the phantom materials and phantom images is shown in Fig. 1(b). The authors concluded that, aside from the image spatial resolution, changes in the MRI sequence parameters do not have a significant influence on the outcome of texture analysis. Even changes in the sequences between 1.5 and 3 T systems were not a critical factor in the outcome.

**Figure 2:**
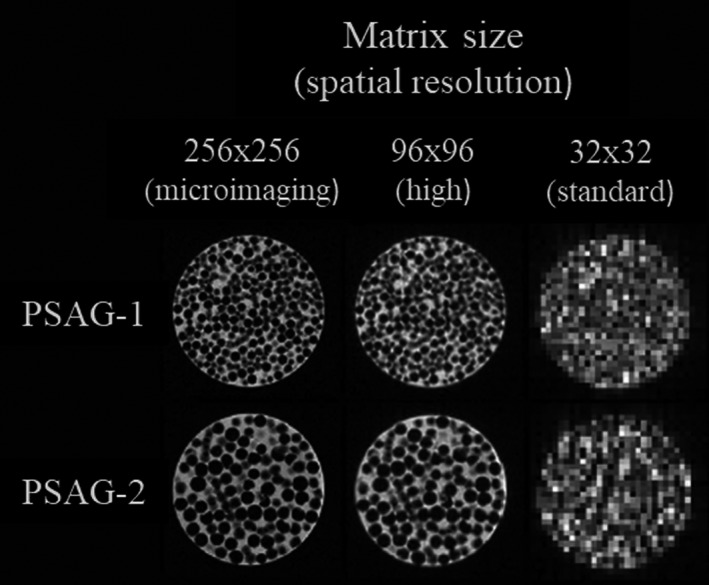
Axial view of texture patterns simulated with polystyrene spheres and agar gel. Spheres diameters used were 1.25–2.0 mm (PSAG‐1) and 2.0–3.15 mm (PSAG‐2). MR images were acquired with different matrix sizes. Pixel sizes were 0.88, 0.098, and 0.018 mm^2^ for standard clinical resolution, high clinical resolution, and micro‐imaging resolution, respectively. Adapted from Fig. 1 in Mayerhoefer et al.^31^, ^32^

A variant of the phantom concepts above was presented by Jirák et al.^30^ In this study, different nodular patterns were modeled by filling three polyethylene tubes with a mixture of polystyrene spheres and agar solution (PSAG). The PSAG phantoms (Fig. 2) produced relaxation times that were in the range of those of biological tissues. The authors performed texture analysis in a 7 T MRI system and five 1.5 T MRI systems. Furthermore, the long‐term stability of the phantom was evaluated over the period of 12 months, when stored at (7 ± 2)°C with periodic 4.7 T MR spectroscopy measurements. The authors demonstrated that the designed objects allow easy texture modeling, suitable for quality control and interscan comparison of texture analysis. However, the authors stated that the phantom classification by texture analysis parameters was not accurate when spheres with diameters below the resolution of the systems were used. In those cases, the differences in textural features values can be attributed to different technical factors (e.g., magnetic field, type of scanner, gradient quality, coils, and differences in scanner’s technology and the time of phantom measurements).

Mayerhoefer et al. also used two PSAG phantoms with different polystyrene sphere diameters (Fig. 2) to evaluate the influence of variation in MRI acquisition parameters and image interpolation on texture analysis results.^31^, ^32^ The images were acquired using a 3 T whole‐body MRI scanner (MEDSPEC S300, Bruker Medical, Ettlingen, Germany), equipped with a dedicated, actively shielded microimaging gradient insert. Good discrimination between the two phantoms was found at microimaging resolution (256 × 256; 0.014 mm^2^ pixel size) and high clinical resolution (96 × 96; 0.098 mm^2^ pixel size), for five texture feature classes. However, at the standard clinical resolution (32 × 32; 0.88 mm^2^ pixel size), rates of misclassification increased considerably.

In summary, all of the mentioned heterogeneity phantoms for MRI are based on embedding structures of solid materials into an agar gel. This concept seems to be suitable for the simulation of heterogeneous structures. However, the limitations of such phantoms are the requirement of specific temperature and humidity conditions for storage, to ensure the standardized use of phantom for repeated measurements and in multicenter trials. A pivotal conclusion that can be drawn from the studies mentioned above is that system resolution is a critical factor when designing phantoms. Using phantom components, such as spheres or pores with sizes below the image resolution, seem to be suboptimal for the simulation of heterogeneities in the context of textural analysis.

## 3 CT Imaging Phantoms

Computed tomography imaging employs x rays to measure the attenuation properties of the investigated object. Therefore, materials with different attenuation coefficients are used in a single phantom to generate CT images with good contrast.^33^ In this sense, a broad spectrum of materials is suitable for CT phantoms. However, one of the challenges for CT phantoms is to find phantom materials, which mimic the x‐ray attenuation properties of the human tissues.^34^, ^35^

**Figure 3:**
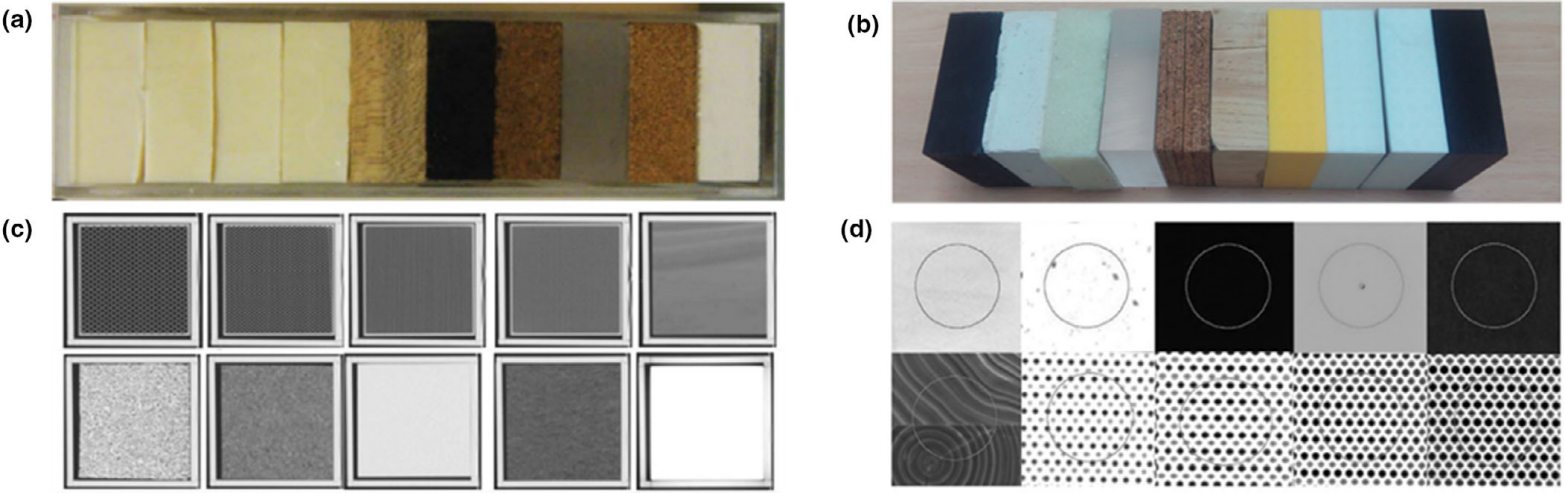
(a,b) Credence Cartridge Radiomics phantoms. Each phantom is composed of ten blocks from different materials. (c,d) Corresponding computed tomography images showing the texture patterns simulated. Adapted from Figs. 1 and 2 in Mackin et al.^36^ and Fig. 1 in Berenguer et al.^38^ [Color figure can be viewed at http://wileyonlinelibrary.com]

In terms of the simulation of texture patterns, Mackin et al. report the design and development of the credence cartridge radiomics (CCR) phantom to test the intra‐ and interscanner robustness and reproducibility of radiomic features.^36^ The CCR phantom comprises ten cartridges (blocks of 10.1 × 10.1 × 3.2 cm^3^) placed in an acrylic case [Fig. 3(a)]. Each cartridge was made from different materials to produce a wide range of radiomics feature values. Four cartridges were 3D printed using acrylonitrile butadiene styrene (ABS) plastic. The inner part of these cartridges consisted of honeycomb patterns with air filled holes of sizes of approximately 6.0, 1.4, 1.0, and 0.9 mm, making the materials 20%, 30%, 40%, and 50% filled, respectively. A block of sycamore wood provided a natural, directional texture. Two cartridges were composed of cork (standard and high density). The eighth cartridge was composed of rubber with a density of 0.93 g/cm^3^ and a speckled texture. The ninth cartridge was a solid block of composite material mixed with a bonding agent. This cartridge presented the highest average density, 1.5 g/cm^3^, and barely visible texture. The last cartridge was made of solid polymethyl–methacrylate (acrylic) with a density of 1.1 g/cm^3^ and very little texture.

The phantom was scanned in 17 different CT scanners from different vendors (General Electric Healthcare, Philips Healthcare, Siemens Healthineers, and Toshiba Medical Systems) using an on‐site chest protocol and standard reconstruction parameters. Two sets of radiomic features were extracted and evaluated: first, entropy and conventional values, such as mean, median, and standard deviation of Hounsfield units (HU); and second, busyness, coarseness, complexity, contrast, and texture strength. It was found that the rubber cartridge had a mean attenuation of −69 HU compared to −54 HU reported for nonsmall cell lung cancer (NSCLC) tumors. After normalization of the feature values to material and scan, none of the scans presented feature values that fell outside two standard deviations from the mean. However, the authors conclude that manufacturer and pixel spacing may affect feature values, even though the images were resampled to 1 mm.

In a similar study, the same CCR phantom was scanned in eight different CT scanners from three different manufacturers to evaluate the dependence of CT radiomic features on voxel size and number of gray levels (NGL).^37^ Resampling of CT phantom image sets to uniform voxel size increased the robustness of 42 out of 213 features studied, thereby suggesting resampling of all image sets to a preselected voxel size as a way to eliminate dependencies introduced by voxel volume or the number of voxels in the ROI.

The use of a similar phantom with 10 cartridges (10 × 10 × 3 cm^3^) from different materials to simulate textural patterns was reported by Berenguer et al.^38^ The selected materials were rubber, plaster, polyurethane, PMMA, cork, wood, and polylactic acid cartridges with a total percentage from 20% to 50% of air content with respect to the solid part. The phantom and corresponding CT images are shown in Figs. 3(b) and 3(d). The phantom was scanned with five different CT systems, and feature extraction was performed on the circular ROIs placed in each material. The reproducibility of the radiomic features was reported to be dependent on the material. For example, high reproducibility of radiomic features (151 of 177 presented COV < 10%) was obtained from the images of the wood cartridge, contrary to the low reproducibility observed for polyurethane (28 of 177 features). Differences between scan manufacturers were reported to be negligible.

**Figure 4:**
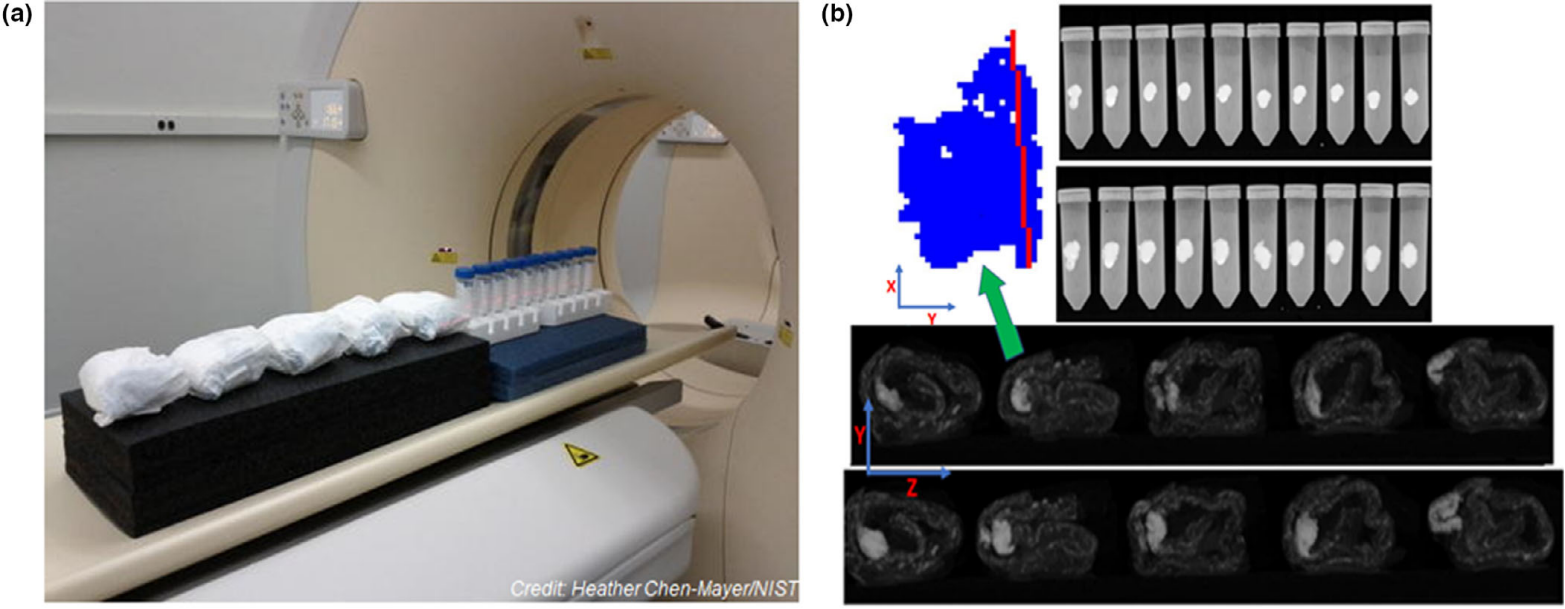
(a) Positioning of vials and diapers used as heterogeneous phantoms for CT scans. (b) Orthographic sagittal projections of the vials (top) and diapers (bottom). The images in the upper part correspond to samples with 2 mL injected water, and the lower images represent the samples after a nominal 4 mL injection of water. Segmentation from one of the diapers (indicated by the green arrow) is shown in blue color. The red line corresponds to the length calculated using RECIST.^8^ Axis Y in the figure corresponds to the direction of gravity in its natural orientation, and axis Z to the scan axis. Taken from Fig. 1 in Levine et al.^39^ [Color figure can be viewed at http://wileyonlinelibrary.com]

The National Institute of Standards and Technology (NIST) reported the use of ten vials containing different amounts of sodium polyacrylate powder and five diapers to simulate random shapes and growth of tumors.^39^ For the measurements, all the 15 objects were placed in a single row [Fig. 4(a)]. A first scan was acquired as a baseline. Furthermore, 2 mL of deionized water was injected into each sample. The samples were weighed and scanned twice. This process was repeated for four consecutive times, adding 0.5 mL of water per time [Fig. 4(b)]. Both approaches allowed to create objects with arbitrary boundaries. However, the model obtained with the diapers was more heterogeneous than those visualized in the vials. The authors developed a predictive model for the assessment of mass and changes in mass with respect to the volume and RECIST‐based measures. Prediction of mass or changes in mass showed a better correlation with changes in volume rather than with RECIST length.

The reviewed CT imaging phantoms present simple designs for simulation of tumor heterogeneities, either textural patterns or heterogeneous sizes and volumes. The simple design of a CCR phantom and its insensitivity to specific storage conditions allow its easy use for interscanner comparison even in multicenter trials. The phantom concept based on vials and diapers with an introduced liquid offers a simple method for evaluation of changes in tumor volume and size over time. However, once the objects are prepared, they cannot be used again, and the reproducibility is limited. Therefore, this approach seems not suitable for longitudinal studies and multicenter trials.

## 4 PET and PET/CT Imaging Phantoms

The principle of PET imaging is the detection of gamma rays (511 keV) originating from the annihilation of positrons with electrons within the examined object. Positron emitters with short half‐life such as 18F are labelled to specific biological molecules and injected into the patients. Depending on the carrier molecule, the radioisotope is distributed across different body tissues, providing physiological information from the region of interest. Therefore, a critical requirement for the design of PET imaging phantoms is the feasibility to simulate radiotracer activity distributions similar to those expected in clinical PET studies. Moreover, PET phantoms should be easy to handle, requiring short preparation times and allowing reproducibility and traceability of quantitative results for the desired imaging application. The compartment materials, should also have low photon attenuation coefficients and be as similar as possible to the human body tissues.

**Figure 5:**
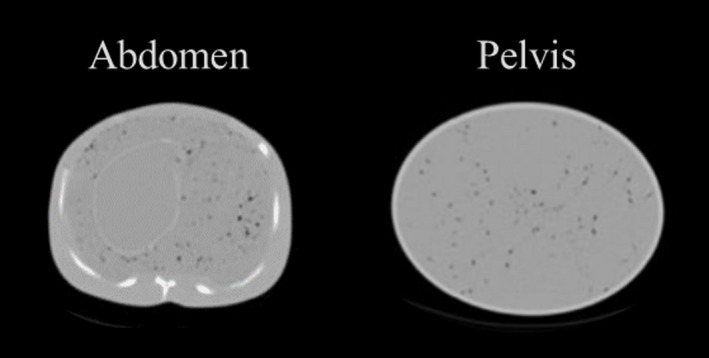
Abdomen and pelvis computed tomography images of whole‐body phantom compartments filled with open cell foam material and radioactive [18F]‐water solution. The simulated heterogeneous pattern of the soft tissues arises from air bubbles suspended in the foam. Adapted from Fig. 2 in Kadrmas et al.^40^

To simulate heterogeneous soft tissue textures similar to those found in patient scans, Kadrmas et al. proposed the use of a cell foam inside a phantom.^40^, ^41^ The foams were inserted into the thorax and pelvis compartment of a whole‐body phantom. When filling the phantoms with a radioisotope, the pores create slightly nonuniform regions (Fig. 5). However, although the model seems to be suitable for analyzing tissue heterogeneities, the authors only report on lesion detectability under heterogeneous background conditions. No characterization of the texture patterns was reported.

**Figure 6:**
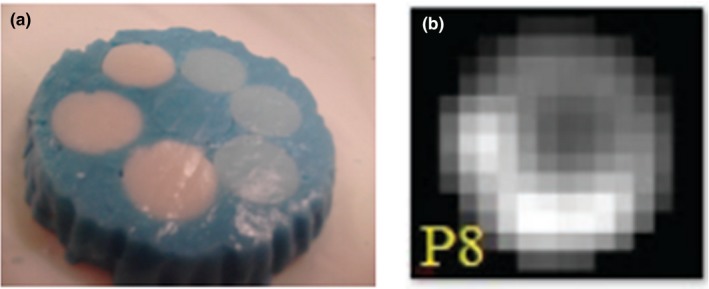
(a) Example of alginate phantom containing circular regions with different activity concentrations and (b) its corresponding positron emission tomography image. Adapted from Fig. 1 in Carles et al.^42^ [Color figure can be viewed at http://wileyonlinelibrary.com]

Carles et al. reported the use of alginate to create heterogeneous phantom inserts and evaluated the effects of respiratory motion in the texture feature analysis.^42^ Combinations of alginate with four different activity ([18F]FDG) concentrations and different geometry arrangements were used to build 28 lesions with a diameter of at least three times the system’s resolution in terms of full‐width‐half‐maximum (FWHM). An activity concentration similar to the one observed in lung cancer patients was ensured during the phantom building process (Fig. 6). The lesions were placed in a phantom filled with a known activity concentration. A Medical QUASAR^™^ respiratory motion phantom^43^ and platform were used to simulate respiratory motion.^43^ Gated, ungated, and static phantom scans were acquired with a PET/CT system. Three different segmentation methods were used separately for feature extraction. Eight texture features (four first order and second order each) were used to quantitate heterogeneity in the simulated lesions. Although volume underestimation was observed for the PET‐based segmentation methods compared with the CT‐based approach, a strong linear correlation was found for most of the evaluated features independently of the segmentation method applied, indicating reliability for their prognostic use.

**Figure 7:**
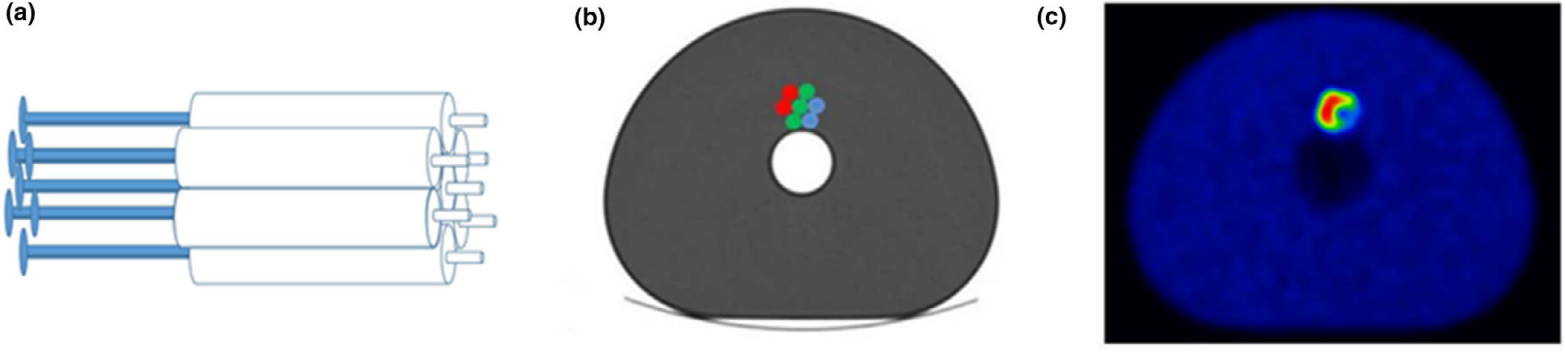
(a) Heterogeneous phantom insert consisting of seven plastic syringes to be filled with radioactivity solutions. (b) Schematic axial view of the insert placed within the NEMA IQ phantom. (c) Example of attenuation corrected positron emission tomography image. Copyright © 2016 Forgacs et al.^15^ [Color figure can be viewed at http://wileyonlinelibrary.com]

Forgacs et al. reported on the use of a heterogeneous phantom insert placed in the NEMA IQ phantom^44^ for PET scans.^15^ The insert (Revolver) consists of seven syringes, as seen in Fig. 7. Red, green, and blue dots correspond to an activity [18F] FDG concentration of 80, 40, 20 kBq/mL while the background activity was 5 kBq/mL. Positron emission tomography/CT scans were performed in three different systems from Philips Healthcare, GE Healthcare, and Siemens Healthineers, and reliability of heterogeneity parameters was assessed from the acquired images. Four of 27 parameters, namely entropy, contrast, correlation, and coefficient of variation, were reported as robust with respect to variables such as the segmented volume. This criterion was fully met when volumes of at least 25–30 mL were used. The sensitivity of the heterogeneity parameters was also evaluated by filling four and three syringes of the insert with 11C‐ and 18F‐solutions, respectively. Then, several PET scans were acquired over time in the same system resulting in different activity ratios due to the difference in half‐life between 11C and 18F. The values of the four features were plotted as a function of time. Here, each feature presented a different level of robustness to measure the degree of heterogeneity.

**Figure 8:**
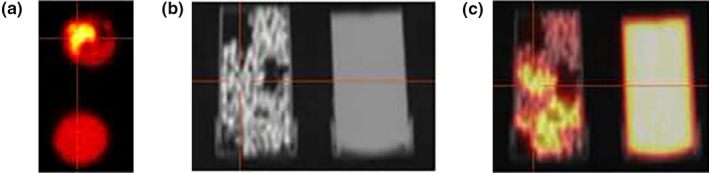
Example of the (a) positron emission tomography (PET), (b) computed tomography (CT), and (c) fused PET/CT images of the heterogeneous sieve phantom. Gel sieves are embedded in solutions with different radioactivity concentrations and then placed in cylindrical containers to simulate heterogeneous patterns. Adapted from Fig. 2 in Presotto et al.^45^ [Color figure can be viewed at http://wileyonlinelibrary.com]

Another phantom concept to simulate tumor‐like heterogeneous radioactivity distributions consists of cylindrical containers filled with silica gel molecular sieves (Heterogeneous Sieve Phantom, HSP).^45^ For this, the same amounts of molecular sieves were embedded in three water solution containers (inner diameter: 32.5 mm, length: 60 mm, volume: 50 ml) with different 18F activity concentrations. Once the sieves absorbed the liquid, the average activity concentrations were 3.6, 7.1, and 21.5 kBq/mL. Mixtures of the sieves were placed in four cylindrical probes to create heterogeneous regions with different spatial radioactivity distributions. PET/CT acquisitions of the phantom were performed with a GE Discovery‐690 PET/CT system (Fig. 8). From 39 texture features extracted and evaluated, 21 were recommended for discrimination of heterogeneous patterns. These include a set of 6, 8, 6, and 1 feature from the gray level run length matrix (GLRLM), gray level size zone matrix (GLSZM), gray‐level co‐occurrence matrix (GLCM), and gray level difference matrix (GLDM), respectively.

**Figure 9:**
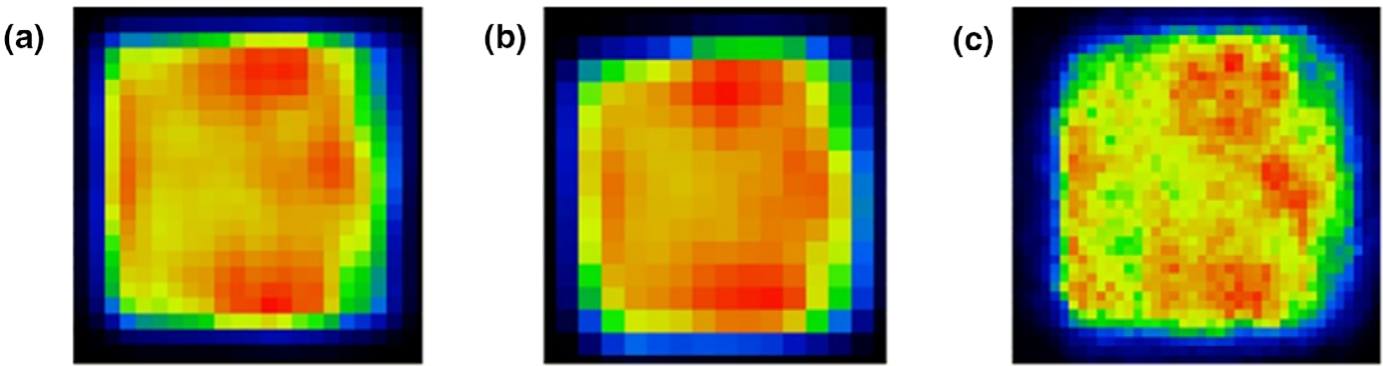
Three‐dimensional heterogeneous lesions simulated with a radioactive point source controlled moved by a robotic arm within the field of view of the imaging system. Images acquired in three different positron emission tomography (PET) scanners: (a) GE Discovery MI PET/computed tomography (CT), (b) Mediso AnyScan PET/CT, (c) Mediso nanoScan PET/magnetic resonance imaging. © 2019 Forgacs et al.^24^ [Color figure can be viewed at http://wileyonlinelibrary.com]

Recently, a different method to mimic heterogeneous PET activity distributions was proposed.^24^ It consists of a robotic arm able to produce precise and reproducible movements along a 3D plane. A calibrated sealed 22Na point source was attached to one of the sides of the arm, and by controlled movements, different activity distributions patterns were drawn (See Fig. 9). Two clinical PET/CT scanners (GE Discovery MI and Mediso Anyscan) and a preclinical nanoScan PET/MRI (Mediso Ltd.) were used for image acquisition. It was noticed that the use of sealed long half‐life radioactivity sources may help to not only ensure a low change in the activity during the measurements but also save phantom preparation time prior to the image acquisition. However, depending on the structure desired, the simulation of specific sizes or patterns can be time consuming.

### 4.1 3D printed PET(/CT) imaging phantoms

Currently, new phantom materials and building techniques are under investigation. Good performance of 3D printing in creating imaging phantoms for different clinical applications has been reported in previous works.^2^, ^46^ These findings indicate the feasibility of using 3D printed models for the development of reproducible and standardized phantoms, thus, permitting the assessment of site‐specific differences for tumor heterogeneity analysis.

**Figure 10:**
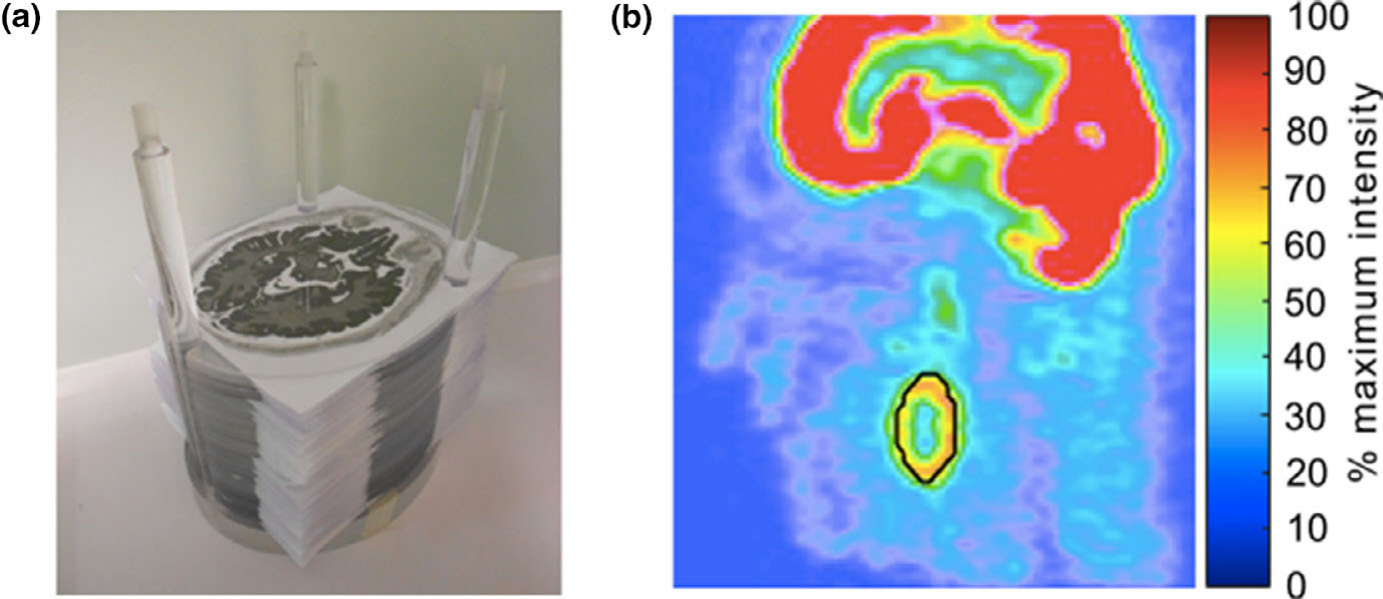
(a) Partly assembled head and neck phantom, containing heterogeneous lesions printed with radioactive ink on paper sheets. (b) Positron emission tomography image of the phantom presenting a necrotic spheroidal lesion. The ground truth is shown in black. © 2015 Berthon et al.^47^ [Color figure can be viewed at http://wileyonlinelibrary.com]

For example, Berthon et al. reported the construction of subresolution sandwich (subS) phantoms for modeling heterogeneous and irregular radiotracer uptake in head and neck cancer patients.^47^ [18F]FDG radioactive ink and a modified conventional printer were used to generate printouts on A4 paper sheets, representing 2‐mm‐spaced slices of modelled head and neck uptake. These printouts were stacked between 2‐mm‐thick PMMA oval sheets (120 in total). All together was assembled using one plastic support Fig. 10(a). Different irregular and spheroidal lesions, including heterogeneous patterns were printed to test the performance of automatic segmentation algorithms for tumor delineation. An example of a necrotic spheroid lesion is shown in Fig. 10(b). The phantom was scanned with a GE 690 Discovery PET/CT system immediately after assembling. An on‐site clinical protocol for whole‐body diagnostic PET was used for the phantom acquisition. The total time from the filling of the printer cartridge to the starting of the phantom scan was 70 min. Of note, this printing technique using radioactive ink was introduced in earlier works.^48^

**Figure 11:**
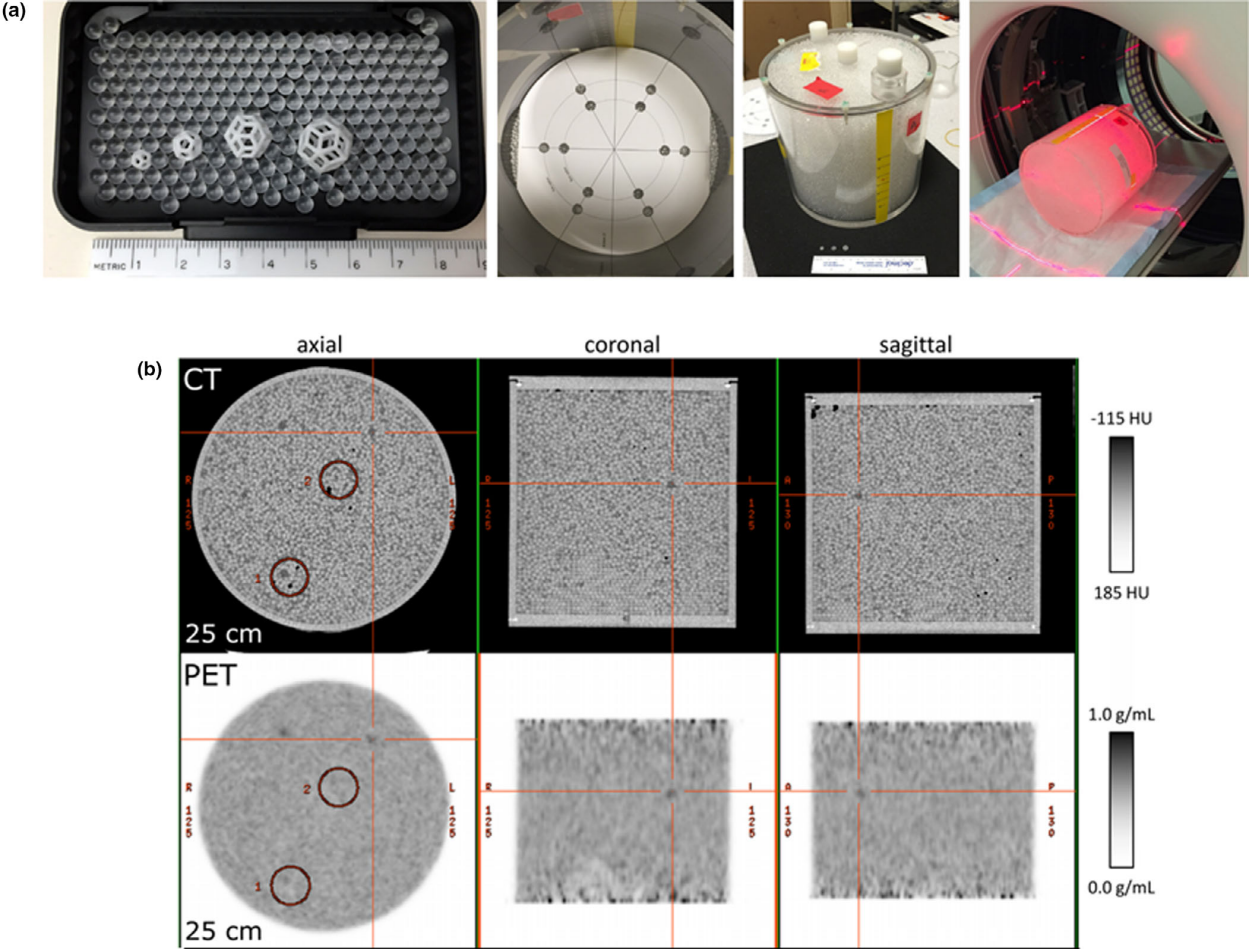
(a) Left to right: Acrylic spheres and four sizes of three‐dimensional–printed features, cylindrical phantom partly filled with acrylic spheres and template for positioning of the features, assembled heterogeneous phantom ready to be filled with radioactive solution and positioning of the phantom for the positron emission tomography (PET)/computed tomography (CT) scans. (b) CT and PET images of the heterogeneous phantom. The intersection of the red lines indicates one of the inserted features. © 2016 Wollenweber et al.^49^ [Color figure can be viewed at http://wileyonlinelibrary.com]

In another study, Wollenweber et al. present a phantom design for assessing lesion detectability in PET.^49^ The phantom includes hollow 3D printed dodecahedral nylon inserts and a heterogeneous background composed of acrylic spheres. The components are embedded in a plastic cylinder compartment filled with [18F]FDG and one drop of detergent as a surfactant. Activity concentration in the hollow features was higher (3:1) than on average in the background, in which the radioactive solution fills the spaces between the spheres Fig. 11. In this study, the phantom was used to evaluate lesion detectability. No characterization of heterogeneity was performed.

**Figure 12:**
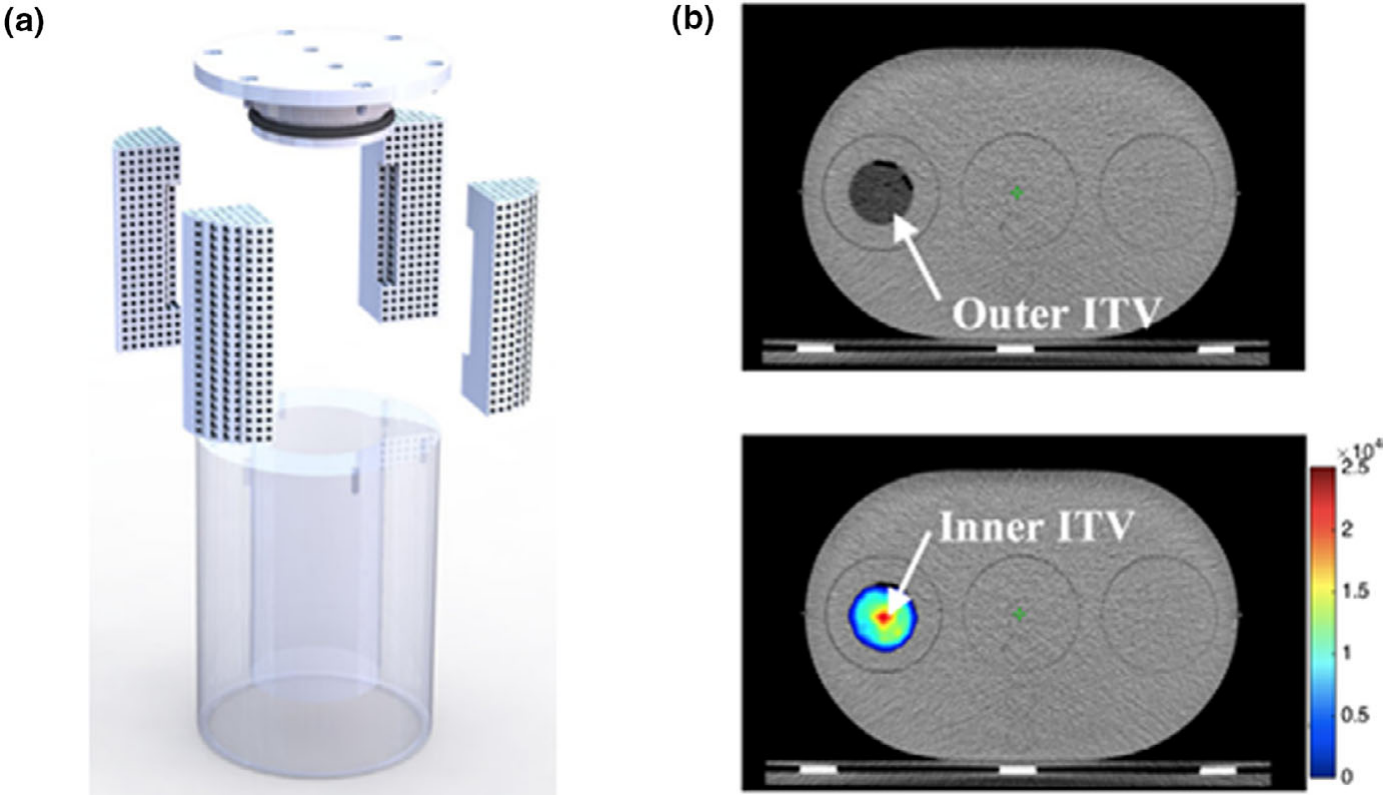
(a) Phantom components: acrylic cylinder, the outer porous region with the hollow inner part, and sealing cap. (b) Corresponding computed tomography (CT) (top) and positron emission tomography/CT fused (bottom) images of the phantom filled with 18F‐FDG. Adapted from Figs. 1 and 6 in Cerviño et al. © 2017 American Association of Physicists in Medicine.^50^ [Color figure can be viewed at http://wileyonlinelibrary.com]

Cervinio et al. reported on a simple design and manufacturing process of a 3D printed phantom insert to simulate heterogeneous radiotracer uptake as observed in lung tumors.^50^ The insert, printed with ABS‐P430 thermoplastic material, was composed of two regions; the outer part was formed by four porous wedges which fill 50% of the outer volume. The four porous parts together generate an inner hollow cylindrical region. This arrangement provides an activity concentration ratio of 2:1 between the inner and outer parts Fig. 12. The insert was placed in a QUASAR^™^ multipurpose body phantom, used as the main compartment. The feasibility of the phantom for four‐dimensional PET/CT quality control, including treatment planning and delivery in simultaneous‐integrated boost (SIB) intensity‐modulated radiotherapy, was demonstrated.

It is important to note that the two prior approaches do not require the filling and mounting of individual subcompartments. Thus, the preparation time is reduced, which is a potential advantage over commonly used phantoms with fillable compartments, such as the NEMA IQ phantom for quality control of PET/CT systems.

**Figure 13:**
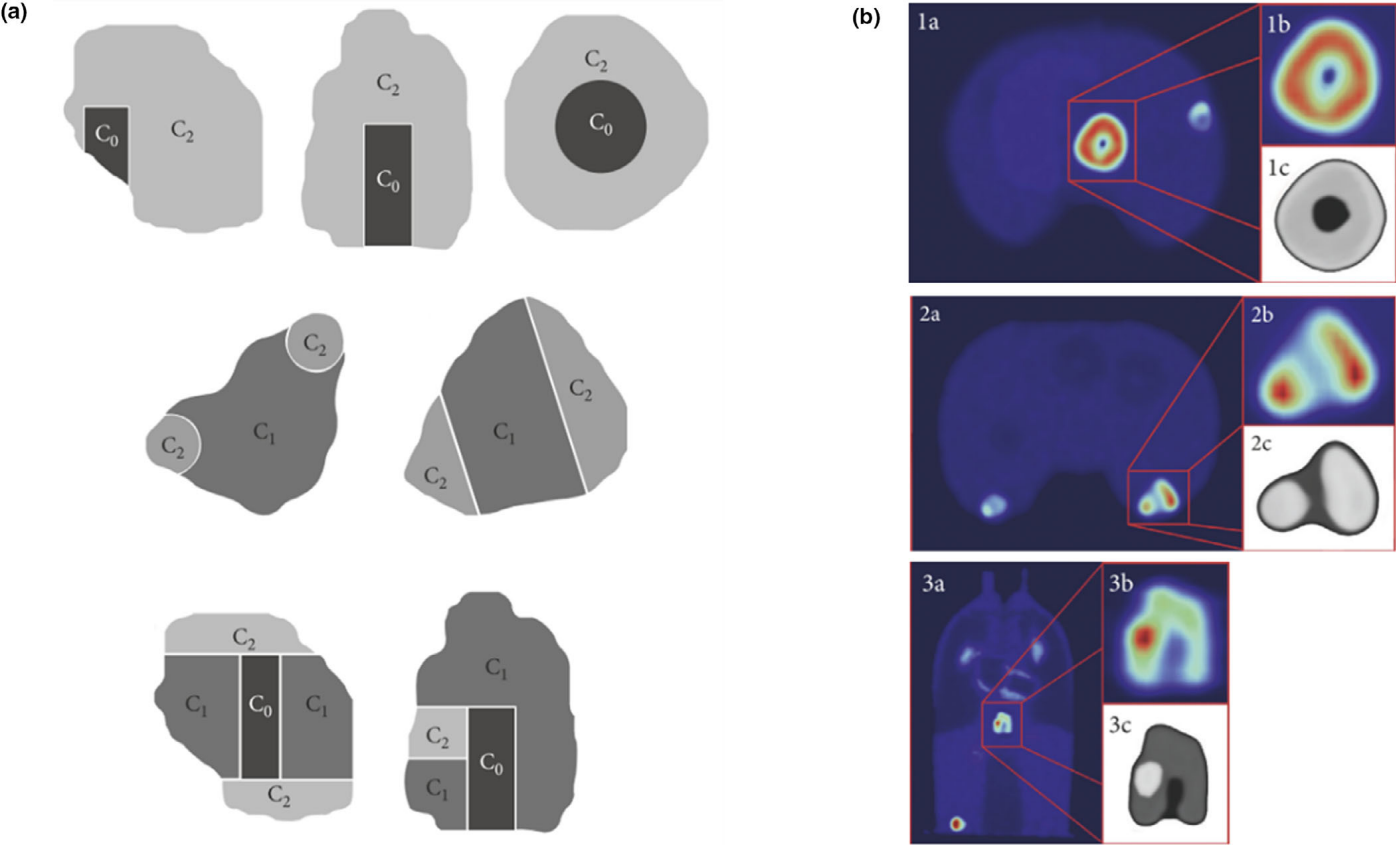
(a) Schemes of different simulated lesions: necrotic (top), heterogeneous (middle), and heterogeneous with necrosis (bottom). C0, C1, and C2 correspond to nonradioactive gel, low concentration, and higher, respectively. (b) Positron emission tomography images of three simulated heterogeneous lesions. Copyright © 2018 Francesca Gallivanone et al.^51^ [Color figure can be viewed at http://wileyonlinelibrary.com]

In a recent study, lesions from oncological patients were segmented and 3D printed as shell compartments, which were filled with [18F]FDG radioactive gels.^51^ Plastic filaments of 3 mm diameter (Renkforce PLA300 Plastic PLA 3 mm) were used to imprint the compartments. The lesions, presenting different shapes and uptake distributions Fig. 13, were placed within the anthropomorphic Alderson thorax phantom for PET scans. For a more realistic patient PET scan, each compartment of the phantom (e.g., liver, lungs, thorax, breast) was filled with a different activity concentration. The authors report a phantom preparation time of about 2 h. Morphological and statistical texture features were extracted from each segmented lesion. Thirty texture features were found to be robust for radiomic analysis. Only three of these features (run‐length non‐uniformity, run percentage, and large zone emphasis) were reported to be representative of heterogeneous PET uptake and reproducible compared with the gold standard for heterogeneity index used by the authors.

**Figure 14:**
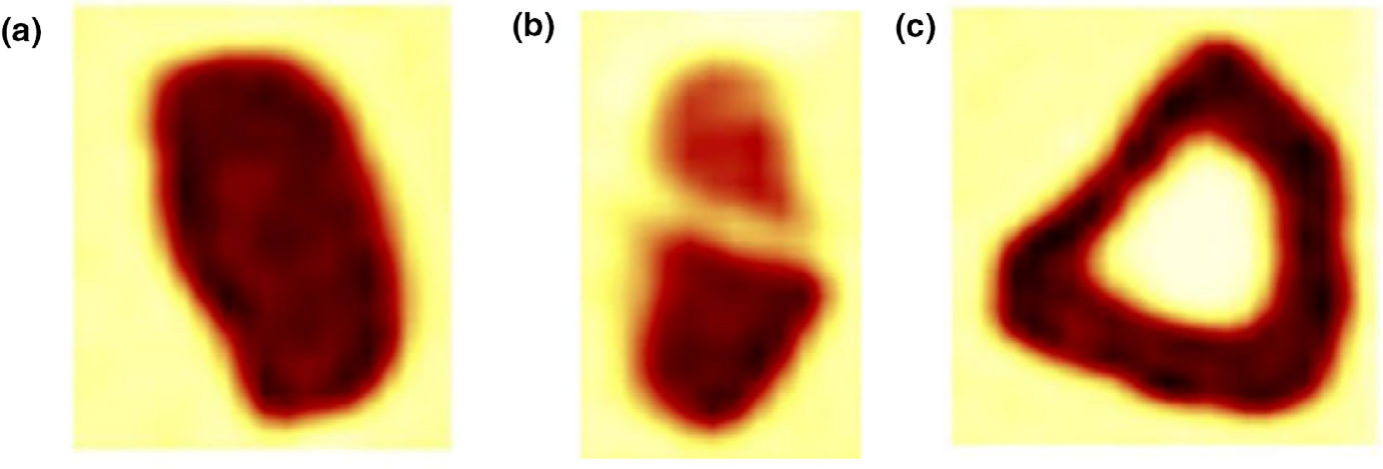
Positron emission tomography images of the (a) uniform, (b) heterogeneous, and (c) necrotic simulated lesions. ©2018 Pfaehler et al.^16^, ^17^ [Color figure can be viewed at http://wileyonlinelibrary.com]

Pfaehler et al. report on the use of 3D printed shells obtained from the segmentation of nonsmall‐cell lung carcinoma (NSCLC) from real patients. Three simulated lesions (homogeneous and heterogeneous uptake) are filled with different activity concentrations of an aqueous solution of [18F]FDG. For PET scans, the 3D printed lesions were placed in the NEMA IQ phantom to be filled with a specific background activity concentration. In a single center study, all phantom scans were acquired with a Siemens Biograph mCT‐40 PET/CT system.^16^ The authors evaluate the influence of reconstruction algorithms, noise, discretization, and delineation methods in the reproducibility of PET radiomic features. Two hundred and forty‐six radiomic features (19 morphological, 3 local‐intensity indexes, 18 statistical, and 206 textural) were calculated from each segmented volume (see Fig. 14) obtained by PET‐based and CT‐based methods. The study demonstrated the high sensitivity of the [18F]FDG radiomic features to most of the evaluated parameters in terms of dimensionality reduction and repeatability. Some features, such as the grey‐level nonuniformity run length and the run length nonuniformity were identified to be repeatable and insensitive to the discretization step. Furthermore, in a multicenter study, the phantom was scanned in six PET/CT systems.^17^ Reconstruction settings given by the European Association of Nuclear Medicine Research Ltd (EARL^52^) accreditation program were applied. The authors evaluated the influence of the EARL‐compliant image reconstructions on the consistency of radiomic features and concluded that EARL‐compliant reconstructions can be used to harmonize a wide range of radiomic features across different settings.

As can be noticed, there is a significant amount of efforts on phantom designs for simulation of tumor heterogeneities in PET and PET/CT. First attempts reported on the use of reticular foams embedded in radioactive solutions, which is a similar concept as used in MR imaging phantoms. Therefore, these phantoms may be usable in MRI as well. Similarly, phantoms based on radioactive alginate or silica gel models may be suitable for multimodality use. However, these phantoms are limited by their poor reusability after radioactive decay, and thus, are not suitable for multicenter trials. Another building concept for PET phantoms is based on fillable compartments. For example, Forgacs et al. proposed to use syringes as compartments inserted into a NEMA IQ phantoms and filled with different radioisotopes to reach a time‐variant activity distribution. The use of fillable compartments enables a reproducible use of the phantom. However, these concepts are often challenged by extended preparation times needed to fill multiple compartments with solutions of varying activity concentration. A completely different concept is based on “painting” a 3D activity distribution into the field of view of a PET scanner by moving a point source to the predefined position using a robotic arm. This enables the simulation of different heterogeneous lesions in a highly reproducible way.

Three‐dimensional printing technology bears a high potential for development of reproducible imaging phantoms with a wide spectrum of materials compatible with different imaging modalities. In the reviewed studies, 3D printing was used to generate simple heterogeneous structures as well as realistic models based on segmentations of real tumor images. However, except the printing of radioactive materials, 3D printing is used to create realistic fillable compartments or structures which are inserted into radioactive solutions similar as discussed already above.

## 5 Summary And Discussion

**Figure 15:**
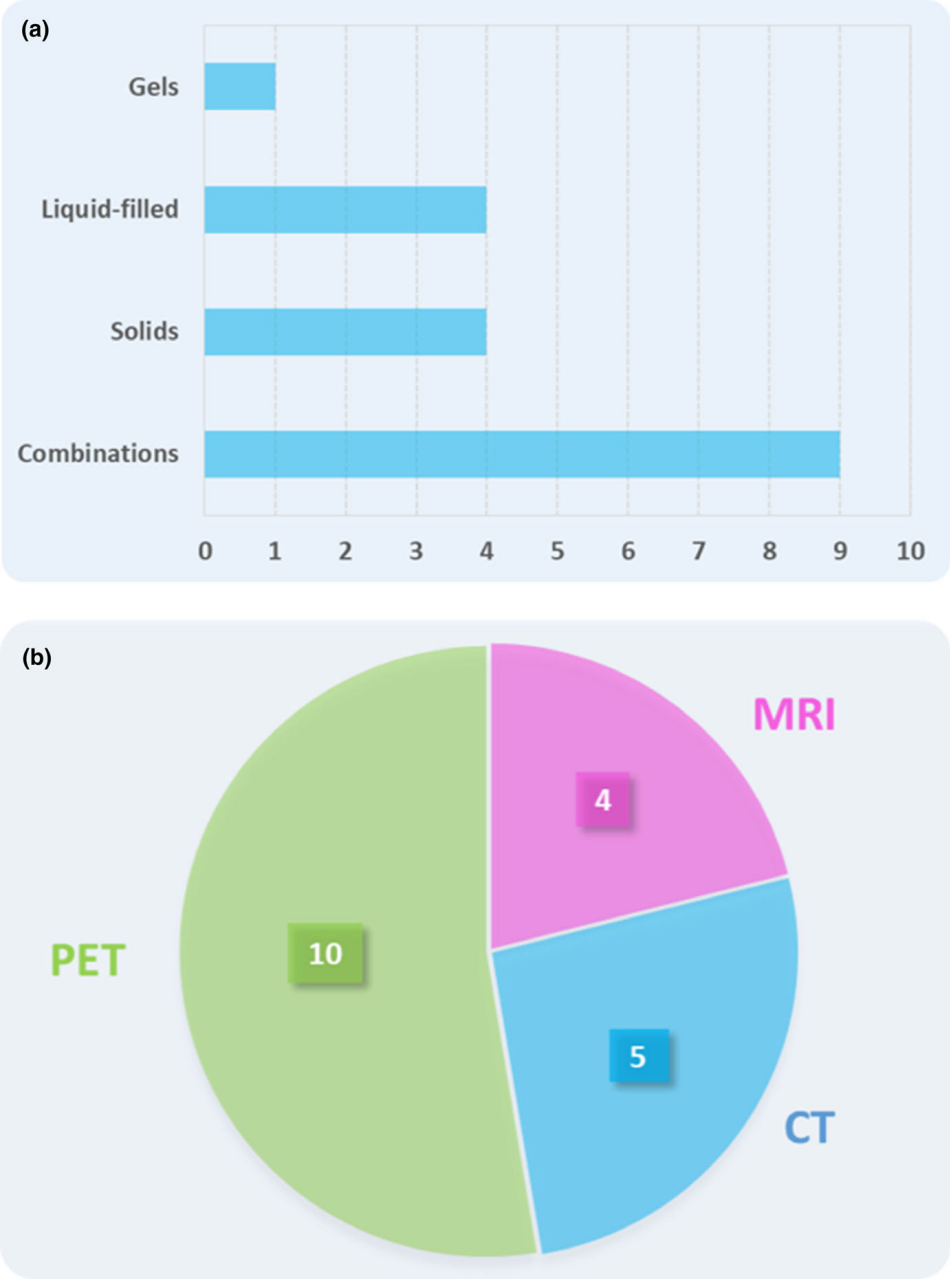
(a) Number of reports that presented a specific type of phantom/phantom insert regarding the materials used to simulate the tumor heterogeneities. (b) The number of times each imaging modality (either dedicated or as part of a hybrid imaging system) was used for phantom scans within the reviewed reports. [Color figure can be viewed at http://wileyonlinelibrary.com]

Physical imaging phantoms provide the means to ensure the proper functioning of the imaging systems while also serving as a reference for establishing reliable quantitation methods for diagnosis, grading, and follow‐up in patient studies. With the adaption of advanced quantitation measures aiming at characterization of tumor heterogeneities, new challenges evolved by means of the construction of phantoms suitable to reflect heterogeneous structures. Several phantoms tackling these challenges have already been introduced for different imaging modalities. As expected, most frequently, water‐filled phantoms were used for PET imaging, while gel‐based and solid phantoms were employed for MRI and CT, respectively Table 1. However, in more than half of the reviewed publications, two or more materials (liquid, gel or solid) were used to create object heterogeneities. A summary of commonly used phantom materials is shown in Fig. 15. From the reported heterogeneous phantom inserts, it was observed that most of the approaches present thick walls, which is not the case in real patient tumors. Considering this factor, the wall‐less models proposed by Carles et al.^42^ and Wollenweber et al.^49^ are good examples of a more realistic tumor heterogeneity simulation.

**Table 1:** Characteristics of the reviewed studies reporting on phantoms/phantom inserts for the simulation of heterogeneities. NA: Not applicable.

Publication (yr)	Materials for heterogeneous phantom/insert	Building technique	Compartment materials	Imaging modality	Radioactive source	Heterogeneity characterization	Multicenter study
Lerski et al. (1998)^28^	Reticulated foams, agarose gel, and glass beads	Handcrafted	Pyrex test tubes	MR (1 T)	NA	Texture analysis	NO
Jirák et al. (2004)^30^	Polystyrene spheres, agar solution	Handcrafted	Polyethylene test tubes	MR (3 T, 7 T)	NA	Texture analysis	NO
Mayerhoefer et al. (2009)^31^, ^32^	Polystyrene spheres and agar solution	Handcrafted	Polyethylene test tubes	MR (3 T)	NA	Texture analysis	NO
Waugh et al. (2011)^29^	Reticulated foams, and 2% agarose solution doped with 0.2% gadolinium	Handcrafted	Polyethylene test tubes	MR (1.5 T, 3 T)	NA	Texture analysis	YES
Mackin et al. (2015)^36^	Acrylonitrile butadiene styrene (ABS) plastic, sycamore wood, cork, rubber, and composite material mixed with a bonding agent	Handcrafted, and 3D printing	Acrylic rectangular cube	CT	NA	Texture analysis	YES
Shafiq‐Ul‐Hassan et al. (2017)^37^	ABS plastic, sycamore wood, cork, rubber, and composite material mixed with a bonding agent	Handcrafted, and 3D printing	Acrylic rectangular cube	CT	NA	Texture analysis	YES
Levine et al. (2017)^39^	Sodium polyacrylate powder, diapers, and water	Handcrafted	Polyethylene test tubes for the sodium polyacrylate powder	CT	NA	Assessment of mass, changes in mass, changes in volume, and RECIST length	NO
Berenguer et al. (2018)^38^	Rubber, plaster, polyurethane, Polymethyl methacrylate (PMMA), cork, wood, and polylactic acid (PLA)	Handcrafted, and 3D printing	Acrylic rectangular cube	CT	NA	Texture analysis	YES
Kadrmas et al. (2009)^40^	Cell foams, radioactive solution	Handcrafted	Anthropomorphic thorax phantom (Radiology Support Devices Inc., Long Beach, CA), and an elliptic cylinder pelvis phantom (Data Spectrum)	PET(/CT)	[18F]FDG	No characterization of heterogeneous patterns	NO
Berthon et al. (2011)^47^	Radioactive ink, paper sheets	Modified common printer	Polymethyl methacrylate (PMMA) sheets and PMMA support	PET(/CT)	[18F]FDG	Delineation of simulated lesions by different segmentation methods	NO
Carles et al. (2016)^42^	Radioactive alginate	Handcrafted	Wall‐less heterogeneous lesions placed in a Medical Quasar respiratory motion phantom	PET(/CT)	[18F]FDG	No characterization of heterogeneous patterns	NO
Wollenweber et al. (2016)^49^	Geometrical nylon inserts, acrylic spheres, radioactive solution	3D printing, handcrafted	Cylindrical ACR PET phantom	PET(/CT)	[18F]FDG	No characterization of heterogeneous patterns	NO
Forgacs et al. (2016)^15^	Plastic syringes, radioactive solutions	Hand crafted	NEMA IQ phantom	PET(/CT)	[18F]FDG, 18F, and 11C	Texture analysis	YES
Cervinio et al. (2017)^50^	ABS‐P430 thermoplastic material, radioactive solution	3D printing	Acrylic cylindrical inserts and the multipurpose body QUASAR phantom	PET(/CT)	[18F]FDG	4D PET/CT quality control, treatment planning, and dosimetry in SIB radiotherapy	NO
Presotto et al. (2018)^45^	Silica gel molecular sieves, radioactive solutions	Handcrafted	Cylindrical containers. No mentioned material	PET(/CT)	[18F]FDG	Texture analysis	NO
Gallivanone et al. (2018)^51^	Plastic filaments (Renkforce PLA300 Plastic PLA 3 mm), radioactive gels	3D printing	Anthropomorphic Alderson Thorax phantom (Radiology Support Devices, Inc.)	PET(/CT)	[18F]FDG	Texture analysis	NO
Pfaehler et al. (2019)^16^	Plastic shells, radioactive solution	3D printing	NEMA IQ phantom^44^	PET(/CT)	[18F]FDG	Texture analysis	NO
Forgacs et al. (2019)^n^	Calibrated sealed radioactive source, robotic arm	Patterns are drawn by automatically controlled movements of the robotic arm	Not applicable	PET	22Na	Texture analysis	YES

One aim of having reference objects for specific imaging applications is their usability across systems and centers, allowing the testing and validation of different imaging methods prior to being applied in patient studies. We observed that a third of the studies included in this manuscript reported on using heterogeneity phantoms for multicenter trials. Nonetheless, for example, for MRI, although only one of the reported phantoms was used in a multicenter trial, the rest of them could also be used for this purpose Table 1. Moreover, most of the phantoms for CT imaging were used in a multicenter trial. However, the reproducibility of the models reported by Levine et al.^39^ to be used across systems and centers is hampered by the lack of control over the water distribution and shape of the simulated lesions. These characteristics were adequate to evaluate changes in mass and volume, but are not suitable for characterization of other heterogeneity features. From the total of PET(/CT/MRI) reviewed studies, only one‐fifth reported on a multicenter trial. The limitation with the use of reticulated foams as well as acrylic spheres embedded in radioactive solutions is the need to change those materials or ensure them to be completely dry before any new phantom image acquisition. Similarly, the radioactive Alginate models, the gel sieves, and the radioactive ink printings cannot be reused once the activity has decayed. In those cases, new objects have to be generated for each PET acquisition.

For the last three PET(/CT) phantom models presented in Table 1, no multicenter study was reported. However, they seem to be reproducible and suitable approaches for comparison of heterogeneity measures across systems and centers.

Table 1 also indicates that half of the studies reviewed did employ standalone imaging modalities for phantom scans, while the other studies reported on the use of hybrid imaging systems. As seen from Fig. 15, PET is the most frequently used (10 studies) imaging modality for tumor heterogeneity evaluation. It includes PET phantom acquisitions either by using a dedicated or a combined PET/CT imaging system. From the total reports involving hybrid imaging scans, only one of them evaluated the heterogeneous patterns by using both the PET and CT components.^50^ For the other PET/CT scans reported, the CT component was mainly used for attenuation correction on the PET images, lesion detectability or lesion segmentation. In the case of the PET/MRI mentioned, only the PET component was used for phantom scans and image analysis.

With the growth of hybrid imaging modalities, new reference objects suitable for validating emerging imaging methods are required. However, the design and development of a suitable object for different modalities are nontrivial. For example, solid materials used for CT imaging do not necessarily present MRI signal–generating properties, which limits their use for MRI. Likewise, some MRI‐compatible materials do not present the desired CT units. Nonetheless, recent studies have explored alternative materials for multimodality imaging.^46^, ^53^, ^54^ These approaches include 3D printing with MR/CT visible materials and phantoms built with graphite–polyurethane mixtures. In terms of suitability for simulating tumor heterogeneity in multimodality imaging, the evaluation of different materials, including synthetic tissue equivalent (STE) ones, has been reported.^55^ In this study, silicone, hydrocarbon, synthetic gelatine, and liquid PVC were visible both with T1 and T2 MR images. Furthermore, hydrocarbon, gelatine, and PVC also presented good CT attenuation and STE properties. Despite the need for suitable materials for CT and MR imaging, the design of fillable compartments for PET imaging is crucial, mainly because of the typically used short half‐life radioisotopes.

Some of the reviewed models for MR imaging might provide, in general, good CT attenuation properties. These objects could also be used for PET imaging by proper mixtures of agar and radioactive solutions. However, the reusability of the phantom for PET imaging would be limited once the activity has decayed. Likewise, most of the presented water‐fillable phantoms for PET(/CT) could be suitable for MR imaging without the need for significant modifications. Only the robotic arm for activity painting proposed by Forgacs et al. is not suitable for cross modality imaging.^24^

Despite all of the reported efforts on the development of heterogeneity phantoms, there appears to be no standardized object that is dedicated for cross modality imaging (PET, CT, MRI). Obviously, such objects would support a single standard for cross modality imaging and, thus, contribute to wide adoption in the imaging community and help facilitate imaging standards. Based on the reviewed reports and the need for standardized physical objects for testing and validation of advanced tumor heterogeneity metrics, some basic requirements for the design and development of PET/CT(MR) imaging phantoms need to be fulfilled. Ideally, these objects should present good MRI contrast in both T1 and T2 MR images, adequate CT attenuation properties, and also allow simulating heterogeneous PET‐tracers activity distribution. Likewise, differences in spatial resolution of the imaging systems need to be addressed. It has been shown that the use of imaging objects with sizes below the system's image resolution could potentially lead to unreliable quantitative metrics. Furthermore, when these objects are intended to be used for multicenter trials, additional characteristics like longevity in composition and shape as well as robustness to transportation conditions have to be ensured. Another critical factor is the level of fidelity and reproducibility reachable with the designed heterogeneous phantoms. Building techniques such as 3D printing have shown good performance and advantages over traditional methods due to their versatility and reproducibility. Furthermore, the exploration of new printable materials with tissue‐like properties bears a high potential for future test objects.

Finally, for more advanced imaging objects, biological factors characteristic of human diseases might have to be considered. Recently, the introduction of organoids for disease characterization seems to be promising for advances in personalized medicine, providing better understanding and modeling of diseases, and thus, contributing to the development of therapeutic tools and drug discovery.^56^ However, although this could be seen as a revolutionary method for a realistic simulation of tumors, some limitations have been already identified. For example, it has been suggested to focus on evaluating and understanding the minimal set of cell types prior to the development and establishment of an organoid model,^57^ for what 3D bioprinting could be a suitable option to do so.^58^ Therefore, it is of critical importance to find a good compromise between a realistic simulation of the tumor heterogeneity and reproducibility of the models in order to make them suitable as potential standardized imaging objects.

## Conflict of Interest

The authors have no conflict to disclose.

## Authors’ contributions

AV, TB, and IR contributed conception and design of the review; AV organized the database and wrote the first draft of the manuscript. All authors contributed to manuscript revision, read and approved the submitted version.
